# An Overview of the Protective Effects of Chitosan and Acetylated Chitosan Oligosaccharides against Neuronal Disorders

**DOI:** 10.3390/md15040089

**Published:** 2017-03-23

**Authors:** Cui Hao, Wei Wang, Shuyao Wang, Lijuan Zhang, Yunliang Guo

**Affiliations:** 1 Institute of Cerebrovascular Diseases, Affiliated Hospital of Qingdao University, Qingdao 266003, China; 18661801189@163.com; 2 Key Laboratory of Marine Drugs, Ministry of Education, Ocean University of China, Qingdao 266003, China; wwwakin@ouc.edu.cn (W.W.); shuyaowang224@126.com (S.W.)

**Keywords:** chitosan, acetylated chitosan oligosaccharides, neuronal disorder, neuroprotection, molecular mechanism

## Abstract

Chitin is the second most abundant biopolymer on Earth and is mainly comprised of a marine invertebrate, consisting of repeating β-1,4 linked *N*-acetylated glucosamine units, whereas its *N*-deacetylated product, chitosan, has broad medical applications. Interestingly, chitosan oligosaccharides have therapeutic effects on different types of neuronal disorders, including, but not limited to, Alzheimer’s disease, Parkinson’s disease, and nerve crush injury. A common link among neuronal disorders is observed at a sub-cellular level, such as atypical protein assemblies and induced neuronal death. Chronic activation of innate immune responses that lead to neuronal injury is also common in these diseases. Thus, the common mechanisms of neuronal disorders might explain the general therapeutic effects of chitosan oligosaccharides and their derivatives in these diseases. This review provides an update on the pathogenesis and therapy for neuronal disorders and will be mainly focused on the recent progress made towards the neuroprotective properties of chitosan and acetylated chitosan oligosaccharides. Their structural features and the underlying molecular mechanisms will also be discussed.

## 1. Introduction

Neurodegeneration, the progressive loss of structure and function including the death of neurons in the central nervous system (CNS), is a major cause of cognitive and motor dysfunction [1]. While neuronal degeneration is well-known in Alzheimer’s and Parkinson’s diseases, it is also observed in neurotrophic infections, neoplastic disorders, prion diseases, multiple sclerosis, amyotrophic lateral sclerosis, stroke, and traumatic brain and spinal cord injuries, in addition to neuropsychiatric disorders and genetic disorders [1,2,3]. A common link among these diseases is observed at a sub-cellular level, such as atypical protein assemblies and induced neuronal death. Chronic activation of innate immune responses that lead to neuronal injury is also common in these diseases [1]. A large collection of evidence indicates that oxidative stress induced by reactive oxygen species (ROS) plays an important role in neurodegenerative diseases [4]. Moreover, high concentrations of glutamate can lead to neuronal injury and cell death through two different mechanisms: an accumulation of oxidative stress [5,6] and a massive influx of extracellular Ca^2+^ [2,7,8]. Thus, the common mechanisms of neuronal damage and neurodegeneration may offer the hope of discovering therapeutics that could treat many neurodegenerative diseases simultaneously. Indeed, chitosan oligosaccharides and their derivatives seem to have effects on different types of neurodegenerative diseases. 

**Figure 1:**
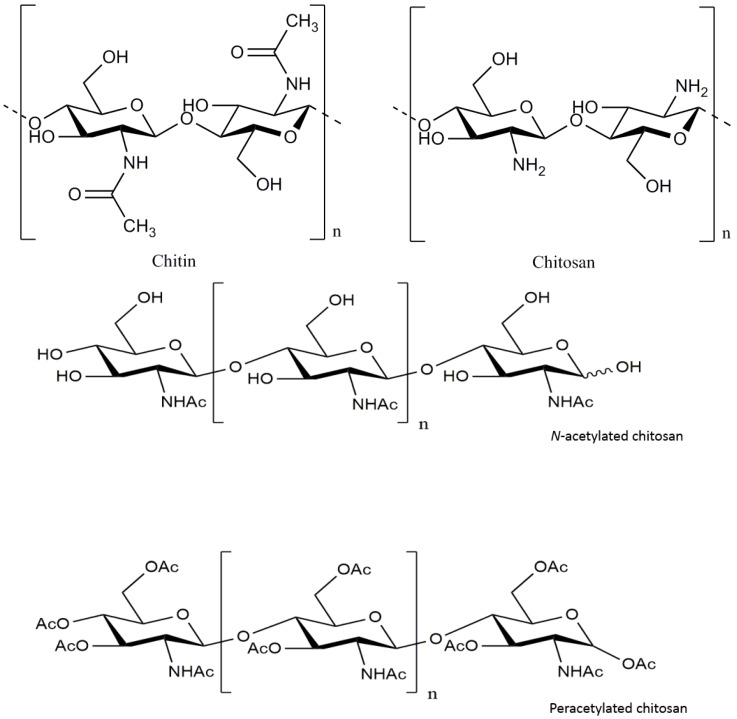
Chemical structure of chitosan and its derivatives.

Chitosan, derived from chitin, is composed of randomly distributed β-(1→4)-linked d-glucosamine and *N*-acetyl-d-glucosamine (Figure 1) [9,10]. Chitin is often present in crustaceans, fungi, yeasts, diatoms sponges, corals, molluscs, and worms [11,12,13,14,15,16]. It is usually obtained by treating the chitin shells of shrimp and other crustaceans with sodium hydroxide [17,18]. Chitosan has received considerable attention as a functional, renewable, nontoxic, and biodegradable biopolymer for diverse applications, especially in pharmaceutics [19], food [20], and cosmetics [21]. In the medical field, chitosan has been developed not only as artificial skin and a wound healing accelerator, but also as a new physiological material due to its antitumor, immunoenhancing, and antimicrobial properties [22].

On average, the molecular weight of commercially produced chitosan is between 3800 and 20,000 Daltons. Chitosan is soluble in acid and relatively insoluble in water. Chitooligosaccharides (COS), i.e., the oligosaccharides of chitosan, are readily soluble in water due to their shorter chain lengths [19]. A large number of studies have shown that the COSs have various biological activities, including antioxidant, antimicrobial, and antitumor activities [19,20]. Recently, it has been reported that the COSs possess good neuroprotective properties, such as β-amyloid and acetylcholinesterase inhibitory activities, anti-neuroinflammation, and anti-apoptosis effects [8,23,24,25,26], which suggest that the COSs and their derivatives might merit further investigation as potential neuroprotective agents against neurodegeneration. 

This review provides an update on the pathogenesis and therapy for neuronal disorders and will mainly focus on the recent progress made towards the neuroprotective properties of chitosan and acetylated chitosan oligosaccharides. The structural features and their underlying molecular mechanisms will also be discussed.

## 2. Update on Pathogenesis and Therapy for Neuronal Disorders

### 2.1. The Pathogenesis of Neuronal Disorders

Neuronal disorders such as Alzheimer’s disease, Parkinson’s disease, amyotrophic lateral sclerosis, and frontotemporal lobar dementia, are among the most pressing problems for aging populations in the world [1,3]. While neuronal degeneration is well-known in Alzheimer’s and Parkinson’s diseases, it is also observed in neurotrophic infections, traumatic brain and spinal cord injuries, stroke, neoplastic disorders, prion diseases, multiple sclerosis, and amyotrophic lateral sclerosis, as well as neuropsychiatric disorders and genetic disorders [1,2,3]. A common link between these diseases is the chronic activation of innate immune responses including those mediated by microglia, the resident CNS macrophages. Such activation can trigger neurotoxic pathways leading to progressive degeneration [1,2]. Moreover, glutamate is one of the major endogenous excitatory neurotransmitters, which plays an important physiological role in the central nervous system [3,7]. However, in a variety of pathological conditions, accumulated high concentrations of glutamate can lead to neuronal injury and cell death through two different mechanisms [2,5,6,7]. One of the mechanisms occurs when glutamate-induced toxicity is mediated by the competitive inhibition of cystine uptake, which leads to oxidative stress [5,6]. Another mechanism presents when the excitotoxicity of glutamate is mediated by several types of excitatory amino acid receptors, resulting in a massive influx of extracellular Ca^2+^ [7,8]. In addition, the toxicity of misfolded protein aggregates is also reported to be responsible for the pathogenesis of Alzheimer’s disease [27,28]. Aβ42 aggregates can cause oxidative stress, [29] and oxidative stress, in turn, increases the β-amyloid cleavage enzyme (BACE-1) activity and Aβ production [30].

Parkinson’s disease (PD) is the second most common neurodegenerative disease, which is characterized by the loss of dopaminergic (DA) neurons in the substantia nigra pars compacta and the formation of Lewy bodies and Lewy neurites in surviving DA neurons, in most cases [31]. Numerous studies have shown that dysfunctional mitochondria may also play key roles in DA neuronal loss [31,32]. Both the genetic and environmental factors that are associated with PD contribute to mitochondrial dysfunction and PD pathogenesis [33]. Neuronal death could be due to metabolic disturbances related to alpha-synuclein accumulation, ubiquitin-proteasome system dysfunction, or oxidative stress [31,34,35]. On the other hand, oxidative stress induced by ROS may also play an important role in neurodegenerative disease, such as Parkinson’s disease [36].

Huntington’s disease (HD) is an autosomal dominant triplet repeat genetic disease, which results in progressive neuronal degeneration in the neostriatum and neocortex, and the associated functional impairments in motor, cognitive, and psychiatric domains [37]. Dopaminergic nigral neurons remain intact in HD and the dopamine level in the HD striatum is higher than normal [38]. Thus, HD is regarded as a relatively dopamine-predominant disease [38], but the mechanism by which this leads to neuronal cell death and the question of why striatal neurons are targeted, both remain unknown [37]. Besides that, prion diseases such as Creutzfeldt-Jakob disease are transmissible fatal neurodegenerative disorders in which infectivity is associated with the accumulation of PrP (Sc), a disease-related isoform of the normal cellular prion protein [39]. The link between PrP (Sc) and neurotoxicity is unclear, and alternative pathological processes need to be considered [39]. New insights into the mechanisms of neurotoxicity in prion diseases support the concept that PrP (Sc) itself is not directly neurotoxic, but neuronal prion propagation results in the production of a toxic intermediate or the depletion of a key constituent [39]. In summary, the pathogenesis process of neuronal disorders is a multi-factor and multi-step process, in which environmental and host factors both play important roles.

### 2.2. Current Treatments and Therapies for Neuronal Disorders

Neurodegenerative diseases are often characterized by the progressive degeneration of the structure and function of the nervous system. The mechanisms and strategies used to protect them against neuronal injury, apoptosis, dysfunction, and degeneration are known as neuroprotection [23,40]. The goal of neuroprotection is to limit neuronal dysfunction or death after CNS injury, in an attempt to maintain the highest possible integrity of cellular interactions in the brain, thus minimizing the disturbance to the neural function [41]. According to the mechanisms of neuroprotection, the neuronal disorders can be treated by using different neuroprotection agents [42,43,44,45,46,47,48,49,50,51,52,53,54,55,56,57], such as antioxidants [42,43] and anti-inflammatory factors [44,45].

Currently, there is no cure for Huntington’s disease. The majority of therapeutics currently used in HD are designed to ameliorate the primary symptomatology of the HD condition itself (psychiatric agents for the control of behavioral symptoms, motor sedatives, cognitive enhancers, and neuroprotective agents), and thus improve the quality of life of the patient [58]. For prion diseases, such as Creutzfeldt-Jakob disease, PrP (Sc) is associated with both pathology and infectivity, and therapeutic approaches to date have largely aimed at preventing its accumulation, but this strategy has only produced modest results in animal models [39]. Passive immunization with anti-prion protein antibodies prevents peripheral prion replication and blocks the progression to clinical disease in peripherally infected mice [39]. Moreover, some disease-modifying therapies have been under development for Alzheimer’s disease, such as BACE inhibitors, and anti-β-amyloid antibodies are in Phase 2 and 3 trials [59]. 

Furthermore, a large collection of evidence indicates that oxidative stress induced by ROS plays an important role in neurodegenerative disease. ROS are normal byproducts of aerobic respiration and their level is strictly controlled by various cellular antioxidant compounds and enzymes, while their overproduction leads to cell death [36]. Accordingly, tackling free radicals offers a promising therapeutic target in neurodegenerative disease. Many categories of natural and synthetic compounds have been reported to possess a neuroprotective activity. However, these synthetic neuroprotective agents are believed to have certain side effects, such as dry mouth, tiredness, drowsiness, sleepiness, anxiety or nervousness, difficulty in balancing, etc. [41]. Therefore, the development of novel anti-neuronal disorder agents with a low toxicity and high efficiency is of great importance.

## 3. The Potential Protective Effects of Chitosan and Its Derivatives against Neuronal Disorders

### 3.1. Potential Applications of Chitosan and Its Derivatives in Alzheimer’s Disease Therapy

Chitosan oligosaccharides (COSs) are a degradation product of chitosan, which is derived from the deacetylation of chitin; the main component of the exoskeleton of crustaceans. Recently, it has been reported that the COSs possess good neuroprotective properties, such as β-amyloid and acetylcholinesterase inhibitory activities, anti-neuroinflammation, and anti-apoptosis effects [23,24,25,26]. Hao and co-workers discovered that the pretreatment of PC12 cells with the peracetylated chitosan oligosaccharides (PACOs) (Figure 1) [8,60] markedly inhibited glutamate-induced cell death in a concentration-dependent manner [8]. PACOs pretreatment significantly reduced lactate dehydrogenase release, reactive oxygen species production, and attenuated the loss of mitochondrial membrane potential. Further studies have indicated that the PACOs inhibited glutamate-induced cell death by preventing apoptosis through depressing the elevation of the Bax/Bcl-2 ratio and caspase-3 activation, which suggested that PACOs might be promising antagonists against glutamate-induced neural cell death [8].

Moreover, it was reported that orally administered COS at 200, 400, or 800 mg/kg doses were effective at reducing the learning and memory deficits in Aβ1-42-induced rats [61]. The neuroprotective effects of COS were closely associated with its ability to inhibit oxidative stress. COS was also shown to suppress the inflammatory response and decrease measures of inflammation via a decrease in the release of proinflammatory cytokines [62]. Thus, COSs have beneficial effects on the cognitive impairments seen in an Aβ1-42-induced model of Alzheimer’s disease via inhibiting oxidative stress and neuroinflammatory responses. In addition, Dai et al. found that COS attenuated Aβ1-42-induced neurotoxicity in the cortical neurons of rats, and COSs may have anti-Aβ fibrillogenesis and fibril-destabilizing properties. Their findings highlight the potential role of COSs as novel therapeutic agents for the prevention and treatment of Alzheimer’s Disease (AD) [61].

### 3.2. The Inhibitory Effects of Chitosan and Its Derivatives against Parkinson’s Disease

Numerous studies have shown that dysfunctional mitochondria may play key roles in DA neuronal loss [31,32]. Both genetic and environmental factors that are associated with PD contribute to mitochondrial dysfunction and PD pathogenesis [33]. Thus, tackling mitochondrial dysfunction offers a promising therapeutic target in neurodegenerative disease. Wang et al. discovered that chitosan (CS) could significantly increase the cell viability and decrease the lactate dehydrogenase (LDH) release induced by Dibutyltin (DBT) in a dose-dependent manner [63]. CS could inhibit cell apoptosis, mitochondrial membrane potential (MMP) disruption, and ROS generation in PC12 cells [63]. Therefore, CS may inhibit DBT-induced apoptosis in PC12 cells through interfering with the mitochondria-dependent pathway [63].

Moreover, COSs were also reported to possess good protective effects against glutamate-induced neurotoxicity in cultured hippocampal neurons [26]. COS pretreatment could inhibit glutamate-induced neuron cell apoptosis in a concentration-dependent manner. COSs depressed glutamate-induced elevation in intracellular calcium concentration Ca^2+^, and antagonized the glutamate-evoked activation of caspase-3 [26]. Thus, COSs may prevent cultured hippocampal neurons from glutamate-induced neuronal cell death by interfering with an increase in Ca^2+^. In summary, chitosan and chitooligosaccharides can also be used for the therapy of Parkinson’s disease.

### 3.3. The Inhibition Effects of Chitosan and Its Derivatives against Huntington’s Disease

Huntington’s disease (HD) is often regarded as a relatively dopamine-predominant disease [38], and there is no effective cure for HD. The majority of therapeutics currently used in HD are designed to ameliorate the primary symptomatology of the HD condition itself, and thus improve the quality of life of the patient [58]. However, the neurotoxicity of glutamate and ROS-induced neuronal damage may also play important physiological roles in the development of Huntington’s disease. Thus, reagents that can inhibit the neurotoxicity of glutamate and ROS may be used for HD therapy.

Xu et al. discovered that chitooligosaccharides possessed protective effects against Cu(II)-induced neurotoxicity in the cortical neurons of rats [64]. Pretreatment with COSs could significantly attenuate the toxicity of Cu(II) to rat cortical neurons in a dose-dependent manner. COSs were found to depress Cu(II)-induced elevation in intracellular reactive oxygen species (ROS). Thus, COSs may protect against Cu(II)-induced neurotoxicity by interfering with the production of intracellular ROS [64]. Therefore, COSs may be used for HD therapy through attenuating the neurotoxicity of glutamate and the production of ROS in neurons.

### 3.4. The Inhibitory Effects of Chitosan and Its Derivatives against Other Neuronal Disorders

Chitosan has been demonstrated to seal compromised nerve cell membranes, thus serving as a potent neuroprotector following acute spinal cord trauma [65]. Cho et al. found that the topical application of chitosan after the complete transection or compression of the guinea pig spinal cord, facilitated the sealing of neuronal membranes in ex vivo tests, and restored the conduction of nerve impulses through the length of spinal cords in vivo, using somatosensory evoked potential recordings [65]. Moreover, chitosan preferentially targeted damaged tissues, serving as a suppressor of reactive oxygen species (ROS) generation, and the resultant lipid peroxidation of membranes, as shown in ex vivo spinal cord samples [65]. Therefore, chitosan treatment can be used as a novel medical approach to reduce the catastrophic loss of behavior after acute spinal cord and brain injuries.

Moreover, Gong and et al. explored the effects of chitooligosaccharides on nerve regeneration after peripheral nerve injuries, and discovered that COS treatment could significantly improve the number of regenerated myelinated nerve fibers, the muscle action potentials, the cross-sectional area of muscle fibers, and the thickness of regenerated myelin sheaths in the nerves [66]. Thus, COSs accelerated peripheral nerve regeneration after a crush injury to the common peroneal nerves of a rabbit. Therefore, the COSs merit further studies as potential neuroprotective agents to improve the peripheral nerve regeneration after an injury [66]. Furthermore, Jiang et al. reported that COS treatment could promote peripheral nerve regeneration with the desired functional recovery in the sciatic nerve crush injury model of a rat, which raises the possibility of developing COS as a potential neuroprotective agent for peripheral nerve repair applications [67]. 

## 4. The Mechanisms of Neuroprotective Effects of Chitosan and Its Derivatives

According to its mechanism, neuroprotection can be categorized into several mechanisms, such as: antioxidant (free radical trapper/scavenger) [42,43]; anti-inflammatory [44,45]; anti-excitotoxic [46]; apoptosis inhibitor [47]; gene expression modulator [48]; ion channel modulator [49,50]; metal ion chelator [51,52]; neurotrophic factor [53,54,55]; matrix metalloprotease inhibitor [56]; and combined mechanism (combining two mechanisms or more) [57].

### 4.1. Anti-Oxidative Stress Action

A large amount of evidence indicates that oxidative stress induced by reactive oxygen species plays an important role in neurodegenerative disease. ROS are normal byproducts of aerobic respiration and their level is strictly controlled by various cellular antioxidant compounds and enzymes, while their overproduction leads to cell death [4]. Accordingly, tackling free radicals offers a promising therapeutic target in neurodegenerative disease. Hao and co-workers indicated that PACOs pretreatment significantly reduced lactate dehydrogenase release and reactive oxygen species production in PC12 cells. Further studies indicated that the PACOs may inhibit glutamate-induced cell death by preventing apoptosis through depressing the elevation of the Bax/Bcl-2 ratio and caspase-3 activation [8]. Moreover, Khodagholi et al. found that chitosan could prevent oxidative stress-induced amyloid β formation in NT2 neuron cells [68], and the chitosan nanoparticles could also effectively, and statistically, reduce damage to the membrane integrity, secondary oxidative stress, and lipid peroxidation. Thus, chitosan may be able to attenuate neuronal damage through inhibiting the production of reactive oxygen species and ROS-induced cell death.

Xu et al. found that COSs showed protective effects against Cu(II)-induced neurotoxicity in the primary cultured cortical neurons of a rat [64]. The toxicity of Cu(II) to cortical neurons was obviously attenuated in a concentration-dependent manner by pretreated COSs. The data derived from lactate dehydrogenase (LDH) release and the Hoechst 33342 assay support the results from the MTT assay. After the 2’,7’-dichlorofluorescin (DCFH) assay, COSs were found to depress Cu(II)-induced elevation in intracellular reactive oxygen species, Therefore, COSs protect against Cu(II)-induced neurotoxicity in primary cortical neurons by interfering with an increase in intracellular reactive oxygen species (ROS) [64].

### 4.2. Suppressing Effect on Abeta Aggregation

β-Amyloid peptide (Aβ), the major component of senile plaques in patients with Alzheimer’s disease (AD), is believed to facilitate the progressive neurodegeneration that occurs in this disease. The β-amyloid (Aβ) peptides can be cleaved from amyloid precursor proteins (APPs) by proteolysis enzymes such as β- and γ-secretase [69,70,71]. In APP proteolysis, it seems that the key enzyme is β-secretase, which is also known as the BACE-1, since it initiates the formation of Aβ [72].

Dai et al. reported that COS could inhibit the formation of Aβ1-42 fibrils and disaggregate preformed fibrils, suggesting that COS may have anti-Aβ fibrillogenesis and fibril-destabilizing properties. Pretreatment with COSs markedly inhibited cell death induced by Aβ exposure, and the ROS generation was also attenuated by COSs [61,73]. Moreover, Je et al. reported that chitosan derivatives could effectively inhibit the activity of BACE-1, and the aminoethyl derivative (AE-chitosan) demonstrated the strongest inhibitory activity compared to other derivatives [74]. Byun et al. indicated that the deacetylated chitosan could obviously inhibit the formation of β-amyloid through blocking the activity of BACE-1 [75]. Thus, the suppression of β-amyloid formation by chitosan and its derivatives may be able to enhance the medications for AD.

### 4.3. Anti-Neuroinflammatory

A growing number of studies are discovering intriguing links between chronic inflammation and a number of neurodegenerative disorders [76]. The neuroinflammation process plays a pivotal role in the initiation and progression of various neurodegenerative diseases [76]. A chronic inflammatory response associated with beta-amyloid (Abeta) and interleukin-1beta (IL-1beta) was reported to be responsible for the pathology of Alzheimer’s disease [77]. Kim et al. discovered that a water-soluble chitosan (WSC) inhibited the production of pro-inflammatory cytokine in human astrocytoma cells activated by Aβ peptide 25–35 (Aβ25–35) and interleukin-1β (IL-1β) [77]. The secretion and expression of pro-inflammatory cytokines, TNF-alpha and IL-6, and the expression of inducible nitric oxide synthase (iNOS), were all significantly inhibited by pretreatment with WSC in human astrocytoma cells [77].

Fang et al. investigated the protective effect and mechanism of chitosan oligonucleotides on retinal ischemia and reperfusion (I/R) injury, and found that pretreatment with COSs, especially at a high dosage, effectively ameliorated the I/R-induced reduction of the b-wave ratio in ERGs and the retinal thickness, and the survival of RGCs at 24 h [78]. COSs decreased the expression of inflammatory mediators, p53 and Bax, increasing Bcl-2 expression and thereby reducing retinal oxidative damage and the number of apoptotic cells. More importantly, COSs attenuated IκB degradation and p65 presence in the retina, thus decreasing NF-κB/DNA binding activity after I/R. In conclusion, COSs prevented retinal I/R injury through their inhibition of oxidative stress and inflammation [78].

### 4.4. Anti-Apoptosis Action

The elimination of cells by apoptosis or programmed cell death is a fundamental event in development, while many human diseases such as acquired immunodeficiency syndrome and neurodegenerative disorders can be directly or indirectly attributed to cell apoptosis [79,80]. In neurodegenerative disorders, apoptosis might be pathogenic, and targeting it might mitigate neurodegenerative disorders [81]. Many researchers have reported that COS and its derivatives may be able to inhibit neuronal cell apoptosis in brain cells.

Wang et al. found that pretreatment with chitosan (CS) significantly increased the cell viability and decreased LDH release induced by DBT in a dose-dependent manner [63]. Meanwhile, DBT-induced cell apoptosis, the disruption of mitochondrial membrane potential (MMP), and the generation of intracellular ROS were attenuated by CS [63]. CS also inhibited the DBT-inducted activation of caspase-9 and -3 at mRNA and protein expression levels. Thus, CS could protect the PC12 cells from apoptosis induced by DBT through the inhibition of the mitochondria-dependent pathway [63]. Moreover, Koo et al. reported that high molecular weight water-soluble chitosan could protect against the cell apoptosis induced by serum starvation in human astrocytes [82]. Thus, the derivatives of chitosan may be able to inhibit neuronal disorders through blocking glutamate-induced neural cell death.

### 4.5. Anti-Excitotoxic Action

Glutamate is one of the major endogenous excitatory neurotransmitters and plays an important physiological role in the central nervous system [3]. However, in a variety of pathological conditions, accumulated high concentrations of glutamate can lead to neuronal injury and cell death, through two different mechanisms. One of the mechanisms occurs when glutamate-induced toxicity is mediated by the competitive inhibition of cysteine uptake, which leads to oxidative stress [5,6]. Another mechanism presents when the excitotoxicity of glutamate is mediated by several types of excitatory amino acid receptors, resulting in a massive influx of extracellular Ca^2+^ [7,8]. Based on both mechanism, it is predictable that proper antagonists would be able to prevent glutamate-induced neural injury and cell death.

Zhou et al. discovered that one chitooligosaccharide (M.W. 800) possessed good protective effects against glutamate-induced neurotoxicity in cultured hippocampal neurons [26]. They found that COS pretreatment could inhibit glutamate-induced cell apoptosis in cultured hippocampal neurons in a concentration-dependent manner. COSs were found to depress glutamate-induced elevation in intracellular calcium concentration Ca^2+^, and could antagonize the glutamate-evoked activation of caspase-3 [26]. Thus, COSs may prevent cultured hippocampal neurons from glutamate-induced neuronal cell death by interfering with an increase in Ca^2+^ and inhibiting caspase-3 activity. Moreover, Dai and co-workers found that COSs may act as inhibitors of Aβ aggregation and this effect shows dose-dependency. The addition of COS could attenuate Aβ1-42-induced neurotoxicity in the cortical neurons of rats [73]. Thus, COS may be able to inhibit neuronal cell damage through interfering with glutamate-induced neurotoxicity, both in vitro and in vivo.

### 4.6. Other Mechanisms

The pathogenesis of AD has been linked to a deficiency in the brain neurotransmitter acetylcholine (ACh) [83,84]. The inhibition of the acetylcholinesterase (AChE) enzyme, which catalyzes the breakdown of ACh, may be one of the most realistic approaches to the symptomatic treatment of AD [83,85,86]. Yoon et al. synthesized COS derivatives with different substitution groups. Among three COS derivatives, diethylaminoethyl-COS (DEAE-COS) has the strongest AChEIs activity, with half maximal inhibitory concentration (IC_50_) values of 9.2 ± 0.33 μg/mL. dimethyl aminoethyl-(DMAE-) and DEAE-COS were identified as competitive AChEIs, according to the Line weaver–Burk plot [87]. These findings suggest that chemical modification will enhance the utilization of COS as AChEIs, and their inhibitory activity depends on the hydrophobic nature of the group that is introduced to them [87].

Furthermore, Gong and co-workers investigated the effects of chitooligosaccharides on nerve regeneration after crush injuries to peripheral nerves, and found that the compound muscle action potentials, the number of regenerated myelinated nerve fibers, the thickness of regenerated myelin sheaths, and the cross-sectional area of tibialis posterior muscle fibers were significantly improved in the nerves that received COS treatment [66]. Thus, the COSs could become potential neuroprotective agents for the improvement of peripheral nerve regeneration after the injury and deserve further consideration [66].

**Figure 2:**
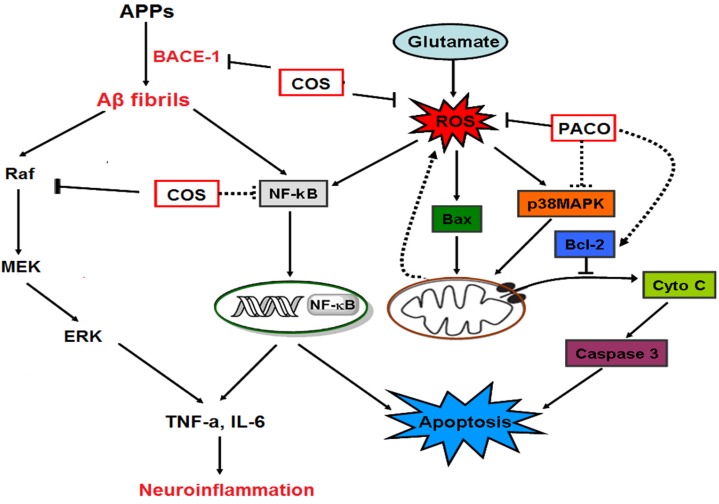
The schematic drawing of anti-neuronal disorder mechanisms of chitosan and its derivatives [8].

In summary, the potential neuroprotective effects of chitosan and its derivatives against neuronal disorders discussed in this paper are summarized in Table 1 and their anti-neuronal disorder mechanisms are presented in Figure 2.

## 5. Progress of the Clinical Studies on Chitosan and Its Derivatives

**Figure 3:**
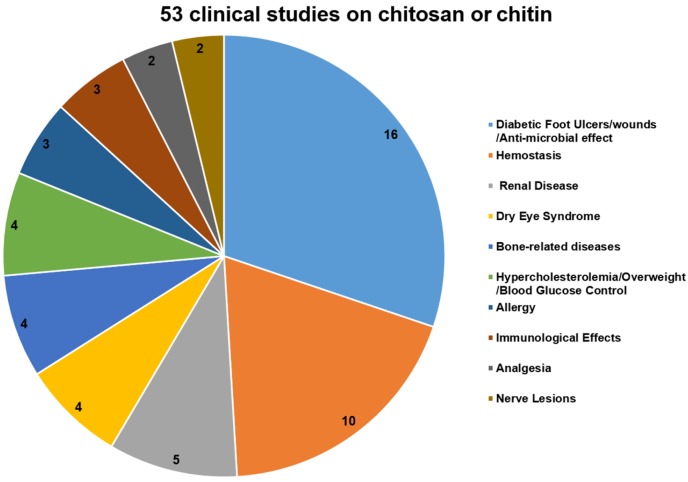
A chart of the clinical studies on chitosan and its derivatives.

To further analyze the potential of chitosan and its derivatives as novel antagonists against neurodegeneration, we also summarize the recent progress in the clinical studies on chitosan and its derivatives as medicinal materials (Figure 3).

As of 8 November 2016, a total of 53 clinical studies on chitosan or chitin were included on the clinicaltrials.gov website [88]. Most of them (16 out of 53) were designed to investigate the therapeutic effects of chitosan-based dressings for wound repair and for minimizing the bacterial re-colonization of wounds, especially in diabetic neuropathic foot ulcers (four cases). Moreover, positively charged chitosan can attract the negatively charged blood cells and platelets to promote clots, so 10 clinical trials were hemostasis studies, including those of a postpartum hemorrhage, dental surgery, and other surgical applications. These two types of clinical trials constitute almost half of the total clinical trials about chitosan and its derivatives (Figure 3). Other clinical trials mainly focused on the application of chitosan in treating chronic kidney disease, dry eye syndrome (DES), bone-related diseases, metabolic diseases, and immune-related diseases (Figure 3).

Furthermore, only two clinical studies were performed to evaluate whether the additional use of a nerve tube in the primary microsurgical repair of traumatic sensory nerve lesions, influences convalescence and functional results. The results showed that chitosan was biocompatible and had positive effects on the survival and orientation of Schwann cells, as well as the survival and differentiation of neuronal cells and the prevention of painful neuromas. Although there has been much research on the neuroprotective effects of chitosan and its derivatives, studies on neuropathy-related clinical trials are rare (Figure 3). Therefore, chitosan and its derivatives merit further investigation in animal experiments or clinical trials as potential anti-neuronal disorder agents.

## 6. Conclusions

Recently, marine polysaccharides and their derivatives have been reported to possess various biological activities, such as antioxidant, antimicrobial, and antitumor activities [19,20]. As one of the bioactive compounds derived from the sea, chitosan and its derivatives have been reported to have good neuroprotective properties, such as β-amyloid and acetylcholinesterase inhibitory activities, anti-neuroinflammation, and anti-apoptosis effects [8,23,24,25,26]. Moreover, the accumulation of chitin fragments may contribute to the dementia of Alzheimer’s disease and chitinase could possibly be used for the treatment of Alzheimer’s disease [89]. Herein, our review presents an overview of the recent progress in research on the neuroprotective effects and mechanisms of chitosan and its derivatives against different neuronal disorders. According to the presented data, it seems that chitosan and its derivatives have the potential to be developed into novel neuroprotective agents in the future. However, further studies are needed in order to explore their activities in animal models and/or clinical trials. Nevertheless, chitosan and its derivatives merit further investigation as potential therapeutic candidates for neurodegenerative disorders.

## Figures and Tables

**Table 1:** Potential neuroprotective effects of chitosan and its derivatives against neuronal disorders.

Specific Polysaccharides	Anti-Neuronal Disorder Effects	Mechanisms	References
Chitosans (CSs)	Anti-Parkinson’s disease; Anti-spinal cord injury	Anti-apoptosis action; Anti-oxidative stress	[63,65,68]
Chitooligosaccharides (COSs)	Anti-Alzheimer’s disease	β-amyloid inhibitory activities; Anti-neuroinflammation; Anti-apoptosis action	[23,24,25,26,61,62,73,74,75]
	Anti-Parkinson’s disease	Anti-exitotoxic action; Anti-apoptosis action	[26]
	Anti-Huntington’s disease	Anti-exitotoxic action; Anti-oxidative stress	[64]
	Anti- nerve crush injury	Promoting nerve regeneration; Anti-neuroinflammation	[66,67,78]
Peracetylated chitosan oligosaccharides	Anti-Alzheimer’s disease; Anti-Parkinson’s disease	Anti-oxidative stress; Anti-apoptosis action	[8]
Water-soluble chitosans	Anti-Alzheimer’s disease	β-amyloid inhibitory activities; Anti-apoptosis	[77,82]
COS derivatives	Anti-Alzheimer’s disease	Anti-AChE and BACE-1 enzyme activities	[74,75,87]

## Abbreviations

Aβ	β-Amyloid peptide
AChE	acetylcholinesterase
AD	Alzheimer’s disease
APPs	amyloid precursor proteins
BACE-1	β-amyloid cleavage enzyme
CNS	central nervous system
COS	chitooligosaccharide
CS	chitosan
DA	dopaminergic
DBT	Dibutyltin
DCFH	2′,7′-dichlorofluorescin
DEAE	diethylaminoethyl
DMAE	dimethyl aminoethyl
HD	Huntington’s disease
IL-1	interleukin-1
iNOS	inducible nitric oxide synthase
LDH	lactate dehydrogenase
MMP	mitochondrial membrane potential
PACOs	peracetylated chitosan oligosaccharides
PD	Parkinson’s disease
ROS	reactive oxygen species

## References

[1] Amor S., Peferoen L.A., Vogel D.Y., Breur M., van der Valk P., Baker D., van Noort J.M. (2014). Inflammation in neurodegenerative diseases—An update. Immunology.

[2] Bleich S., Romer K., Wiltfang J., Kornhuber J. (2003). Glutamate and the glutamate receptor system: A target for drug action. Int. J. Geriatr. Psychiatry.

[3] Choi D.W. (1988). Glutamate neurotoxicity and diseases of the nervous system. Neuron.

[4] Droge W. (2002). Free radicals in the physiological control of cell function. Physiol. Rev..

[5] Murphy T.H., Miyamoto M., Sastre A., Schnaar R.L., Coyle J.T. (1989). Glutamate toxicity in a neuronal cell line involves inhibition of cystine transport leading to oxidative stress. Neuron.

[6] Zablocka A., Janusz M. (2008). The two faces of reactive oxygen species. Postep. Hig. Med. Doswiadczalnej.

[7] Monaghan D.T., Bridges R.J., Cotman C.W. (1989). The excitatory amino acid receptors: Their classes, pharmacology, and distinct properties in the function of the central nervous system. Annu. Rev. Pharmacol. Toxicol..

[8] Hao C., Gao L., Zhang Y., Wang W., Yu G., Guan H., Zhang L., Li C. (2015). Acetylated chitosan oligosaccharides act as antagonists against glutamate-induced PC12 cell death via Bcl-2/Bax signal pathway. Mar. Drugs.

[9] Kumar M.N., Muzzarelli R.A., Muzzarelli C., Sashiwa H., Domb A.J. (2004). Chitosan chemistry and pharmaceutical perspectives. Chem. Rev..

[10] Zargar V., Asghari M., Dashti A. (2015). A Review on Chitin and Chitosan Polymers: Structure, Chemistry, Solubility, Derivatives, and Applications. ChemBioEng Rev..

[11] Brunner E., Richthammer P., Ehrlich H., Paasch S., Simon P., Ueberlein S., van Pee K.H. (2009). Chitin-based organic networks: An integral part of cell wall biosilica in the diatom *Thalassiosira pseudonana*. Angew. Chem. Int. Ed. Engl..

[12] Ehrlich H. (2010). Chitin and collagen as universal and alternative templates in biomineralization. Int. Geol. Rev..

[13] Ehrlich H., Ilan M., Maldonado M., Muricy G., Bavestrello G., Kljajic Z., Carballo J.L., Schiaparelli S., Ereskovsky A., Schupp P. (2010). Three-dimensional chitin-based scaffolds from Verongida sponges (Demospongiae: Porifera). Part I. Isolation and identification of chitin. Int. J. Biol. Macromol..

[14] Bo M., Bavestrello G., Kurek D., Paasch S., Brunner E., Born R., Galli R., Stelling A.L., Sivkov V.N., Petrova O.V. (2012). Isolation and identification of chitin in the black coral *Parantipathes larix* (Anthozoa: Cnidaria). Int. J. Biol. Macromol..

[15] Anitha A., Sowmya S., Kumar P.T.S., Deepthi S., Chennazhi K.P., Ehrlich H., Tsurkan M., Jayakumar R. (2014). Chitin and chitosan in selected biomedical applications. Prog. Polym. Sci..

[16] Wysokowski M., Petrenko I., Stelling A., Stawski D., Jesionowski T., Ehrlich H. (2015). Poriferan Chitin as a Versatile Template for Extreme Biomimetics. Polymers.

[17] Hong K., Meyers S.P. (1995). Preparation and Characterization of Chitin and Chitosan—A Review. J. Aquat. Food Prod. Technol..

[18] Younes I., Rinaudo M. (2015). Chitin and chitosan preparation from marine sources. Structure, properties and applications. Mar. Drugs.

[19] Zhang J., Xia W., Liu P., Cheng Q., Tahirou T., Gu W., Li B. (2010). Chitosan modification and pharmaceutical/biomedical applications. Mar. Drugs.

[20] Shahidi F., Abuzaytoun R. (2005). Chitin, chitosan, and co-products: Chemistry, production, applications, and health effects. Adv. Food Nutr. Res..

[21] Nan W., Sun A. (2003). Application of Chitosan and Oligochitosan in the Field of Cosmetics. Chem. Ind. Eng. Prog..

[22] Bellich B., D’Agostino I., Semeraro S., Gamini A., Cesaro A. (2016). “The Good, the Bad and the Ugly” of Chitosans. Mar. Drugs.

[23] Pangestuti R., Kim S.K. (2010). Neuroprotective properties of chitosan and its derivatives. Mar. Drugs.

[24] Lee S.H., Park J.S., Kim S.K., Ahn C.B., Je J.Y. (2009). Chitooligosaccharides suppress the level of protein expression and acetylcholinesterase activity induced by Abeta25–35 in PC12 cells. Bioorg. Med. Chem. Lett..

[25] Nidheesh T., Salim C., Rajini P.S., Suresh P.V. (2016). Antioxidant and neuroprotective potential of chitooligomers in *Caenorhabditis elegans* exposed to Monocrotophos. Carbohydr. Polym..

[26] Zhou S., Yang Y., Gu X., Ding F. (2008). Chitooligosaccharides protect cultured hippocampal neurons against glutamate-induced neurotoxicity. Neurosci. Lett..

[27] Soto C. (2003). Unfolding the role of protein misfolding in neurodegenerative diseases. Nat. Rev. Neurosci..

[28] Khanam H., Ali A., Asif M., Shamsuzzaman (2016). Neurodegenerative diseases linked to misfolded proteins and their therapeutic approaches: A review. Eur. J. Med. Chem..

[29] Butterfield D.A., Swomley A.M., Sultana R. (2013). Amyloid beta-peptide (1–42)-induced oxidative stress in Alzheimer disease: Importance in disease pathogenesis and progression. Antioxid. Redox Signal.

[30] Chami L., Checler F. (2012). BACE1 is at the crossroad of a toxic vicious cycle involving cellular stress and beta-amyloid production in Alzheimer’s disease. Mol. Neurodegener..

[31] Hu Q., Wang G. (2016). Mitochondrial dysfunction in Parkinson’s disease. Transl. Neurodegener..

[32] Jana S., Sinha M., Chanda D., Roy T., Banerjee K., Munshi S., Patro B.S., Chakrabarti S. (2011). Mitochondrial dysfunction mediated by quinone oxidation products of dopamine: Implications in dopamine cytotoxicity and pathogenesis of Parkinson’s disease. Biochim. Biophys. Acta.

[33] Migliore L., Coppede F. (2009). Genetics, environmental factors and the emerging role of epigenetics in neurodegenerative diseases. Mutat. Res..

[34] Recchia A., Debetto P., Negro A., Guidolin D., Skaper S.D., Giusti P. (2004). Alpha-synuclein and Parkinson’s disease. FASEB J..

[35] Lim K.L. (2007). Ubiquitin-proteasome system dysfunction in Parkinson’s disease: Current evidence and controversies. Expert Rev. Proteom..

[36] Andersen J.K. (2004). Oxidative stress in neurodegeneration: Cause or consequence?. Nat. Med..

[37] Kumar A., Kumar Singh S., Kumar V., Kumar D., Agarwal S., Rana M.K. (2015). Huntington’s disease: An update of therapeutic strategies. Gene.

[38] Spokes E.G. (1980). Neurochemical alterations in Huntington’s chorea: A study of post-mortem brain tissue. Brain.

[39] Mallucci G., Collinge J. (2004). Update on Creutzfeldt-Jakob disease. Curr. Opin. Neurol..

[40] Kostrzewa R.M., Segura-Aguilar J. (2003). Novel mechanisms and approaches in the study of neurodegeneration and neuroprotection. A review. Neurotox. Res..

[41] Tucci P., Bagetta G. (2008). How to study neuroprotection?. Cell. Death Differ..

[42] Pellicciari R., Costantino G., Marinozzi M., Natalini B. (1998). Modulation of glutamate receptor pathways in the search for new neuroprotective agents. Farmaco.

[43] Behl C., Moosmann B. (2002). Antioxidant neuroprotection in Alzheimer’s disease as preventive and therapeutic approach. Free Radic. Biol. Med..

[44] Agnello D., Bigini P., Villa P., Mennini T., Cerami A., Brines M.L., Ghezzi P. (2002). Erythropoietin exerts an anti-inflammatory effect on the CNS in a model of experimental autoimmune encephalomyelitis. Brain Res..

[45] Gao H.M., Liu B., Zhang W., Hong J.S. (2003). Novel anti-inflammatory therapy for Parkinson’s disease. Trends Pharmacol. Sci..

[46] Volbracht C., van Beek J., Zhu C., Blomgren K., Leist M. (2006). Neuroprotective properties of memantine in different in vitro and in vivo models of excitotoxicity. Eur. J. Neurosci..

[47] Yu X., An L., Wang Y., Zhao H., Gao C. (2003). Neuroprotective effect of *Alpinia oxyphylla* Miq. fruits against glutamate-induced apoptosis in cortical neurons. Toxicol. Lett..

[48] Kietzmann T., Knabe W., Schmidt-Kastner R. (2001). Hypoxia and hypoxia-inducible factor modulated gene expression in brain: Involvement in neuroprotection and cell death. Eur. Arch. Psychiatry Clin. Neurosci..

[49] Heurteaux C., Guy N., Laigle C., Blondeau N., Duprat F., Mazzuca M., Lang-Lazdunski L., Widmann C., Zanzouri M., Romey G. (2004). TREK-1, a K+ channel involved in neuroprotection and general anesthesia. EMBO J..

[50] Schwartz G., Fehlings M.G. (2001). Evaluation of the neuroprotective effects of sodium channel blockers after spinal cord injury: Improved behavioral and neuroanatomical recovery with riluzole. J. Neurosurg..

[51] Youdim M.B., Fridkin M., Zheng H. (2004). Novel bifunctional drugs targeting monoamine oxidase inhibition and iron chelation as an approach to neuroprotection in Parkinson’s disease and other neurodegenerative diseases. J. Neural Transm..

[52] Gaeta A., Hider R.C. (2005). The crucial role of metal ions in neurodegeneration: The basis for a promising therapeutic strategy. Br. J. Pharmacol..

[53] Tremblay R., Hewitt K., Lesiuk H., Mealing G., Morley P., Durkin J.P. (1999). Evidence that brain-derived neurotrophic factor neuroprotection is linked to its ability to reverse the NMDA-induced inactivation of protein kinase C in cortical neurons. J. Neurochem..

[54] Moalem G., Gdalyahu A., Shani Y., Otten U., Lazarovici P., Cohen I.R., Schwartz M. (2000). Production of neurotrophins by activated T cells: Implications for neuroprotective autoimmunity. J. Autoimmun..

[55] Akerud P., Canals J.M., Snyder E.Y., Arenas E. (2001). Neuroprotection through delivery of glial cell line-derived neurotrophic factor by neural stem cells in a mouse model of Parkinson’s disease. J. Neurosci..

[56] Woo M.S., Park J.S., Choi I.Y., Kim W.K., Kim H.S. (2008). Inhibition of MMP-3 or -9 suppresses lipopolysaccharide-induced expression of proinflammatory cytokines and iNOS in microglia. J. Neurochem..

[57] Chandrasekaran K., Mehrabian Z., Spinnewyn B., Chinopoulos C., Drieu K., Fiskum G. (2003). Neuroprotective effects of bilobalide, a component of Ginkgo biloba extract (EGb 761) in global brain ischemia and in excitotoxicity-induced neuronal death. Pharmacopsychiatry.

[58] Handley O.J., Naji J.J., Dunnett S.B., Rosser A.E. (2006). Pharmaceutical, cellular and genetic therapies for Huntington’s disease. Clin. Sci. (Lond.).

[59] Selkoe D.J., Hardy J. (2016). The amyloid hypothesis of Alzheimer’s disease at 25 years. EMBO Mol. Med..

[60] Han Z., Zeng Y., Lu H., Zhang L. (2015). Determination of the degree of acetylation and the distribution of acetyl groups in chitosan by HPLC analysis of nitrous acid degraded and PMP labeled products. Carbohydr. Res..

[61] Dai X., Chang P., Zhu Q., Liu W., Sun Y., Zhu S., Jiang Z. (2013). Chitosan oligosaccharides protect rat primary hippocampal neurons from oligomeric beta-amyloid 1–42-induced neurotoxicity. Neurosci. Lett..

[62] Jia S., Lu Z., Gao Z., An J., Wu X., Li X., Dai X., Zheng Q., Sun Y. (2016). Chitosan oligosaccharides alleviate cognitive deficits in an amyloid-beta1–42-induced rat model of Alzheimer’s disease. Int. J. Biol. Macromol..

[63] Wang X., Miao J., Yan C., Ge R., Liang T., Liu E., Li Q. (2016). Chitosan attenuates dibutyltin-induced apoptosis in PC12 cells through inhibition of the mitochondria-dependent pathway. Carbohydr. Polym..

[64] Xu W., Huang H.C., Lin C.J., Jiang Z.F. (2010). Chitooligosaccharides protect rat cortical neurons against copper induced damage by attenuating intracellular level of reactive oxygen species. Bioorg. Med. Chem. Lett..

[65] Cho Y., Shi R., Borgens R.B. (2010). Chitosan produces potent neuroprotection and physiological recovery following traumatic spinal cord injury. J. Exp. Biol..

[66] Gong Y., Gong L., Gu X., Ding F. (2009). Chitooligosaccharides promote peripheral nerve regeneration in a rabbit common peroneal nerve crush injury model. Microsurgery.

[67] Jiang M., Zhuge X., Yang Y., Gu X., Ding F. (2009). The promotion of peripheral nerve regeneration by chitooligosaccharides in the rat nerve crush injury model. Neurosci. Lett..

[68] Khodagholi F., Eftekharzadeh B., Maghsoudi N., Rezaei P.F. (2010). Chitosan prevents oxidative stress-induced amyloid beta formation and cytotoxicity in NT2 neurons: Involvement of transcription factors Nrf2 and NF-kappaB. Mol. Cell Biochem..

[69] Evin G. (2016). Future Therapeutics in Alzheimer’s Disease: Development Status of BACE Inhibitors. BioDrugs.

[70] Lukiw W.J. (2008). Emerging amyloid beta (Ab) peptide modulators for the treatment of Alzheimer’s disease (AD). Expert Opin. Emerg. Drugs.

[71] Okamura N., Suemoto T., Shiomitsu T., Suzuki M., Shimadzu H., Akatsu H., Yamamoto T., Arai H., Sasaki H., Yanai K. (2004). A novel imaging probe for in vivo detection of neuritic and diffuse amyloid plaques in the brain. J. Mol. Neurosci..

[72] Hampel H., Shen Y. (2009). Beta-site amyloid precursor protein cleaving enzyme 1 (BACE1) as a biological candidate marker of Alzheimer’s disease. Scand. J. Clin. Lab. Investig..

[73] Dai X., Hou W., Sun Y., Gao Z., Zhu S., Jiang Z. (2015). Chitosan Oligosaccharides Inhibit/Disaggregate Fibrils and Attenuate Amyloid beta-Mediated Neurotoxicity. Int. J. Mol. Sci..

[74] Je J.Y., Kim S.K. (2005). Water-soluble chitosan derivatives as a BACE1 inhibitor. Bioorg. Med. Chem..

[75] Byun H.-G., Kim Y.-T., Park P.-J., Lin X., Kim S.-K. (2005). Chitooligosaccharides as a novel β-secretase inhibitor. Carbohydr. Polym..

[76] Kim Y.S., Joh T.H. (2006). Microglia, major player in the brain inflammation: Their roles in the pathogenesis of Parkinson’s disease. Exp. Mol. Med..

[77] Kim M.S., Sung M.J., Seo S.B., Yoo S.J., Lim W.K., Kim H.M. (2002). Water-soluble chitosan inhibits the production of pro-inflammatory cytokine in human astrocytoma cells activated by amyloid beta peptide and interleukin-1beta. Neurosci. Lett..

[78] Fang I.M., Yang C.M., Yang C.H. (2015). Chitosan oligosaccharides prevented retinal ischemia and reperfusion injury via reduced oxidative stress and inflammation in rats. Exp. Eye Res..

[79] Twomey C., McCarthy J.V. (2005). Pathways of apoptosis and importance in development. J. Cell. Mol. Med..

[80] Fadeel B., Orrenius S. (2005). Apoptosis: A basic biological phenomenon with wide-ranging implications in human disease. J. Intern. Med..

[81] Vila M., Przedborski S. (2003). Targeting programmed cell death in neurodegenerative diseases. Nat. Rev. Neurosci..

[82] Koo H.N., Jeong H.J., Hong S.H., Choi J.H., An N.H., Kim H.M. (2002). High molecular weight water-soluble chitosan protects against apoptosis induced by serum starvation in human astrocytes. J. Nutr. Biochem..

[83] Tabet N. (2006). Acetylcholinesterase inhibitors for Alzheimer’s disease: Anti-inflammatories in acetylcholine clothing!. Age Ageing.

[84] Terry A.V., Buccafusco J.J. (2003). The cholinergic hypothesis of age and Alzheimer’s disease-related cognitive deficits: Recent challenges and their implications for novel drug development. J. Pharmacol. Exp. Ther..

[85] Ibrahim F., Andre C., Thomassin M., Guillaume Y.C. (2008). Association mechanism of four acetylcholinesterase inhibitors (AChEIs) with human serum albumin: A biochromatographic approach. J. Pharm. Biomed. Anal..

[86] Martinez A., Castro A. (2006). Novel cholinesterase inhibitors as future effective drugs for the treatment of Alzheimer’s disease. Expert Opin. Investig. Drugs.

[87] Yoon N.Y., Ngo D.-N., Kim S.-K. (2009). Acetylcholinesterase inhibitory activity of novel chitooligosaccharide derivatives. Carbohydr. Polym..

[88] Clinical Trials. https://clinicaltrials.gov.

[89] Stern R. (2017). Go Fly a Chitin: The Mystery of Chitin and Chitinases in Vertebrate Tissues. Front. Biosci. (Landmark Ed.).

